# Contribution to the Cross-Border Public Health of Mosquito Control Programs Applied in Evros Prefecture, Greece (2015–2018)

**DOI:** 10.7759/cureus.81126

**Published:** 2025-03-24

**Authors:** Christos F Nanos, Sofia Mainou, Evangelia Nena, Gregory Trypsianis, Theodoros Konstantinidis, Theodoros S Lialiaris

**Affiliations:** 1 Laboratory of Genetics, Department of Medicine, Democritus University of Thrace, Alexandroupolis, GRC; 2 Laboratory of Social Medicine, Faculty of Medicine, Democritus University of Thrace, Alexandroupolis, GRC; 3 Laboratory of Medical Statistics, Department of Medicine, Democritus University of Thrace, Alexandroupolis, GRC; 4 Laboratory of Hygiene and Environmental Protection, Department of Medicine, Democritus University of Thrace, Alexandroupolis, GRC

**Keywords:** adulticiding, greece, larviciding, malaria, mosquitoes, west nile virus

## Abstract

Vector-borne diseases are among the leading causes of death globally. Mosquitoes breeding in open water sources are associated with the transmission of sporadic diseases, and their management differs from species reproducing in urban and peri-urban areas. Invasive species such as *Aedes* and *Culex* mosquitoes pose significant public health challenges. The climatic conditions in the examined area (Evros, North Eastern Greece) in the period between 2015 and 2018 had been challenging, with high temperatures and heavy rainfall having led to significant increases in mosquito populations. The combination of the abovementioned climatic conditions, along with regional topography and migration flows at the borders, can contribute to the resurgence of West Nile virus and malaria cases, which have been sporadically reported. Despite the widespread implementation of larviciding (i.e., the process of controlling insect larvae, primarily mosquitoes, using chemical or biological substances aimed at killing the larvae before they reach their adult form), mosquito nuisance levels remained very high in several areas, causing significant problems to the residents. The recent emergence of the Asian *Aedes albopictus* (known as the “tiger mosquito”) in the Evros Regional Unit is particularly concerning as it can transmit severe and possibly fatal diseases. A cornerstone of future integrated control programs will be systematic entomological surveillance and epidemiological research. In addition, creating an information platform for the entire Greek territory that is continuously updated with relevant data is imperative.

## Introduction

This study aims to evaluate the effectiveness of mosquito control programs implemented in Evros Prefecture between 2015 and 2018, assess their impact on vector-borne disease prevalence, and identify challenges in cross-border public health management. Furthermore, it highlights the importance of climate change, as a severe problem is created when there is heavy rainfall in weather phenomena. Consequently, many mosquito breeding grounds are created because mosquitoes lay their eggs in these specific areas. Therefore, there should be cooperation between mosquito control programs and other public bodies involved, so that all these breeding grounds can be eliminated (e.g., grass cutting, canal cleaning, etc.). Migration flows affect mosquito control programs because some migrants come from areas where they carry diseases (such as malaria, dengue fever, etc.). This results in mosquitoes biting these people and transferring them to other people by biting them. 

It is also well known that mosquitoes are vectors with a significant role in the transmission of pathogens, transmitting severe diseases to both humans and animals [1]. High mosquito populations negatively impact local economies and residents' quality of life [2-3]. Recent increases in West Nile virus cases across Europe, alongside dengue, malaria, and yellow fever, have raised concerns particularly as these diseases, previously controlled or locally have been eliminated in Greece. On the contrary, they have recently re-emerged in neighboring countries [4-5]. Notable cases in Cyprus, Germany, France, and Italy highlight the urgent need for timely and well-coordinated mosquito control programs [6]. Significant scientific attention has been directed toward large water basins, such as the Evros River Delta, due to their suitability as mosquito habitats, as well as border areas accommodating migrants originating from countries endemic to mosquito-borne diseases [7]. In recent years, a significant increase in migration into Greece, from regions where invasive mosquito species are prevalent, was observed [8] coming from regions where invasive mosquito species (e.g., *Aedes albopictus*) are prevalent. This can lead to the potential emergence of previously unknown serious diseases in Greece (e.g. Dengue, Malaria, and Yellow Fever) posing significant epidemiological risks. In high-risk areas and periods of potential outbreak, mosquito management programs are systematically implemented and intensified when necessary [4,9].

The goal of this study was to collect and analyze data from the integrated mosquito control programs implemented by Eastern Macedonia and Thrace Region (PAMTH) in Evros Prefecture between 2015 and 2018 to help understand the issues arising and to prompt improvements in future control measures [10-11].

## Materials and methods

**Figure 1:**
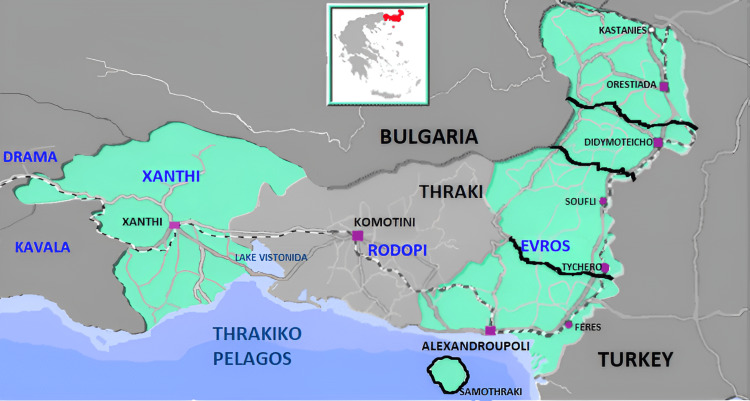
Map of the Evros prefecture (Greece). Image Credits: Nanos CF, Author

Data were collected, focusing on epidemiological data related to mosquito-borne diseases in Evros Prefecture. In addition, statistics from various mosquito control programs across Greece were gathered (Evros included) (see map in Figure 1).

A comparison was made of bibliographic data on insecticides from domestic and international sources [3,11]. In addition, a comprehensive literature review concerning new biocide formulations and their effectiveness against local and non-endemic mosquito species was conducted alongside statistical analyses of epidemiological data regarding mosquito-borne diseases. Resistance studies on endemic and non-endemic mosquito species to biocides were performed based on data collected from public and private agencies involved in mosquito control [5-6,9,12]. Finally, an analysis of results from a monitoring network using Centers for Disease Control and Prevention (CDC) light traps [12] was carried out, along with experimental applications of new technologies (e.g., drones) in the field of mosquito control [13].

Key data were derived from the protocols completed by responsible teams for each mosquito control action annually. Additional information on mosquito-borne diseases was obtained from official sources like the websites of the World Health Organization (WHO - www.who.int), the National Public Health Organization (EODY - www.eody.gov.gr), and the Hellenic Statistical Authority (www.statistics.gr). All data came from the Eastern Macedonia and Thrace Region (PAMTH) Public Health services, after approval for the retrospective processing and publication of the data (Protocol Νο: 19400/96-20.01.2025). For the statistical analyses presented, all sources mentioned above were utilized to compile the information that formed the basis of the tables and charts. The results were pooled for analysis using IBM SPSS Statistics for Windows (IBM Corp., Armonk, NY). Numbers of the three dominant genera (*Aedes*, *Culex*, and *Anopheles*) were calculated by the chi-square test, which was employed to examine differences between samples to assess significant deviations compared with controls (p < 0.05).

## Results

The practical implications of the study were to provide guidance on the timely tendering process of integrated mosquito control programs so that their duration starts from the beginning of April of each year and ends at the end of October. This, of course, depends on the respective weather conditions. In addition, the duration of each mosquito control program should be at least three years, so that their timely start of operation is possible. So, it is necessary that each program is implemented and that there are appropriate state funds.

Overview of the situation in 2015 and 2016

The program, along with the development of various operational maps, was initiated later than expected. This delay resulted in incomplete preparatory field activities within the appropriate timeframe (March-April-May) and a pressing need during the specific period of July to intensify the mosquito spraying applications. Consequently, within the short duration of 23 days that the signed contract was in effect, the marking and geo-referencing of the entire hydrographic network were fully completed. This included all relevant measurements (such as streams, channel lengths, existing rice paddies, etc.) for the entire Evros Prefecture, thereby facilitating the updating of all electronic records for improved operational efficiency. The complexity involved in creating this specific database proved to be a particularly time-consuming process, attributed to the unique geomorphological conditions; however, it emerged as the most functional tool for the organization and assessment of the entire project, particularly concerning ground-based mosquito control spraying operations.

By the end of July, the collection of adult mosquitoes commenced. The teams deployed carbon dioxide traps at 10 designated locations within the northern and southern regions of Evros Prefecture and implemented biweekly sampling at each station. Upon completion of the collection at each site, a count and identification of the genera of the captured adult mosquitoes were conducted. The collected samples were subsequently sent to the European Laboratory for Biological Control, located at the American Agricultural School in Thessaloniki (https://www.ars.usda.gov/office-of-international-research-engagement-and-cooperation/ebcl/ ). By August 31, 2015, a total of 20 adult mosquito sampling events were conducted in the Evros Prefecture, utilizing carbon dioxide traps placed at specific locations. It should be mentioned that in 2015, no West Nile virus cases were reported in Evros nor in the rest of Greece. One case of the virus was recorded in Bulgaria in 2015. On the contrary, in 2014, 10 cases had been documented in neighboring prefectures, eight in Xanthi’s prefecture and two in Rhodope’s prefecture, which became new areas of viral circulation, indicating virus circulation in the area of Thrace and a potential establishment in various species of wild birds, particularly corvids (Figure 1). In Europe and neighboring countries during 2014, various West Nile virus cases were recorded, such as Serbia, Bosnia-Herzegovina, Romania, Israel, Austria, and Italy [10]. 

The timely initiation of the mosquito control program was possible this year, which prevented any operational delays. Throughout the winter of 2015 and into the spring of 2016, sampling was conducted along with 23 adult mosquito control interventions in various locations, such as stable facilities and abandoned buildings, where healthy adults had taken refuge for overwintering. During this year, a significant increase in *Aedes* mosquitoes was observed, except in the Didymoteicho area, which experienced a noticeable decrease due to enhanced larviciding measures. The weather conditions were relatively favorable for the season, and there was an increase in the *Culex* species; however, this was not particularly concerning compared to previous years. One of the significant challenges faced during implementation was limited access to focal points such as drainage channels and streams. Another contributing factor was the lack of road networks, along with the presence of dense vegetation within the streams. It is understood that difficult access to these sites presents obstacles both for sampling and for spraying operations, as it hampers the contact of the insecticide formulation with the sprayed surfaces. At the same time, cleaning the streams plays a crucial role in achieving the best possible results, as existing vegetation can retain waste and hinder spraying teams in their efforts. Unfortunately, it has been frequently observed that wastewater is deposited in stormwater drains, drainage channels, and streams in many settlements. Several communities have constructed concrete drainage channels for stormwater, which often become sites for waste disposal, creating additional breeding habitats.

In October, the Evros Prefecture experienced increased drought and a notable drop in temperature, leading to a reduction in breeding sites. By the end of the month, the only productive sites remaining were those near livestock facilities, such as watering troughs and intentional flooding for irrigating fields, among others. In 2016, as in 2015, no cases of West Nile virus were reported in humans in Greece. However, the virus has established itself in the country, and its circulation is expected to continue, contrary to all forecasts compared to previous years. By contrast, seven cases of malaria were reported in the country, particularly in regions such as Thessaly and the Peloponnese, but not in Thrace. This is noteworthy given the onset of migratory flows from the land borders, which have created several epidemiological challenges in the Evros region. In neighboring countries, two cases of *West Nile virus* were reported in Turkey and another two in Bulgaria [10]. 

For a major understanding, the mosquito control programs for Evros and Thrace, in general, started for the first time in 2014 but were delayed and had minimal organization. Thus, in the year 2015, the competition for the selection of a contractor company was delayed in completing. This resulted in a very short duration (23 days). Consequently, this continued in 2016. The fairly significant unmapped area of ​​mosquito breeding grounds contributed to this, as well as the smaller number of traps operating for the mosquito, where the data for statistical processing was insufficient. The year 2017 was the starting point for a continuous increase in funds, traps, and mosquito capture. In conclusion, a better organizational tool was created for the creation of a database of mosquito control programs for the years 2017-2018.

Overview of the situation in 2017

**Figure 2:**
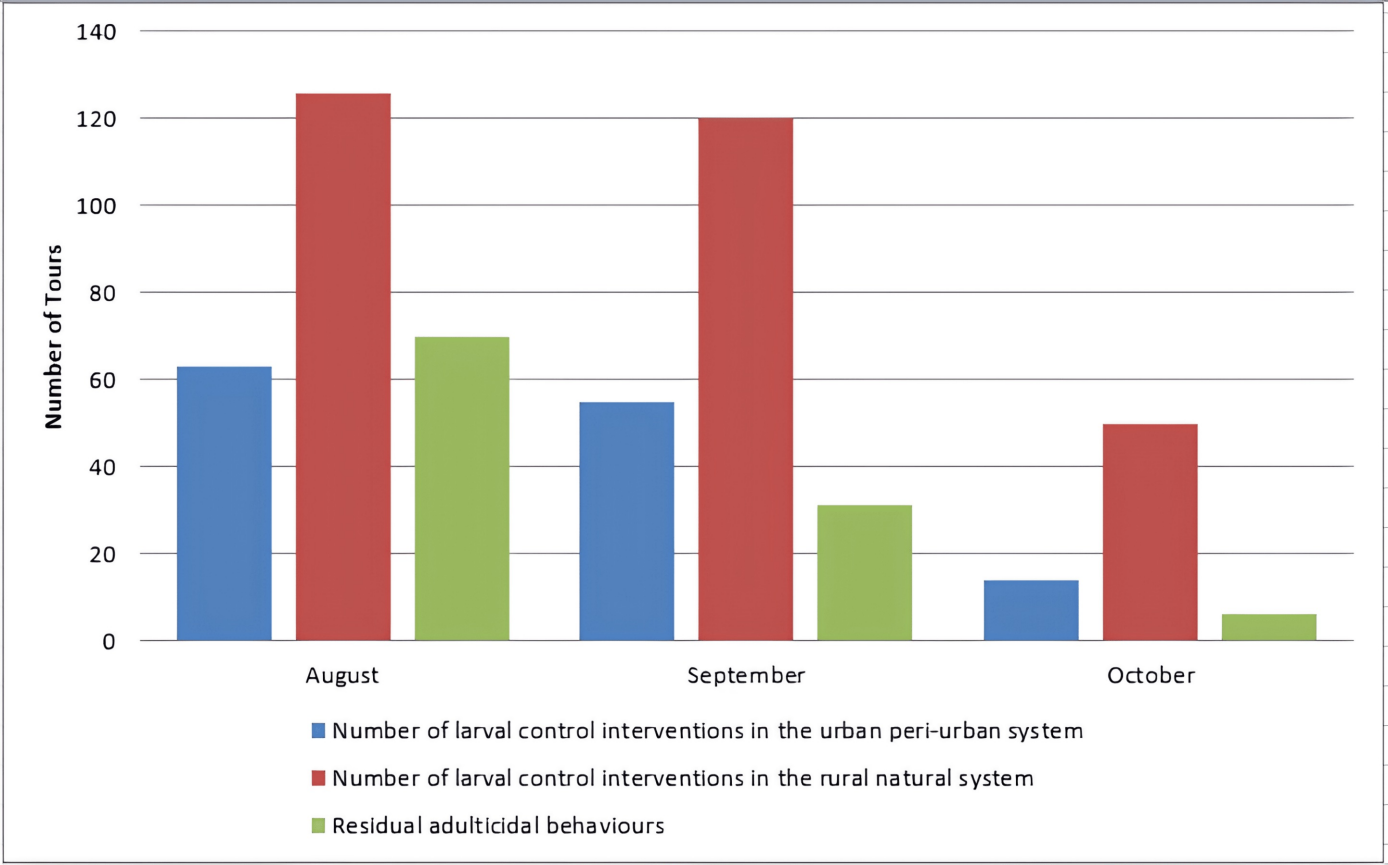
: Graphical representation of spraying interventions for 2017. Image credits: Nanos CF, Lialiaris TS, authors

In 2017, the information presented was significantly more. This is mainly attributed to the gaining experience from conducting mosquito control programs in the region over the previous three years. The result was the gradual improvement of the program and the better handling of certain technical problems. The mosquito control operations in the Evros Prefecture began with the signing of the contract on July 27, 2017, which was significantly delayed until nearly the middle of summer. In addition, there was a doubling of the budget for ground actions in the Evros Prefecture. At the same time, residual adult mosquito spraying was implemented in various settlements, migrant reception centers, and customs facilities, as well as during events, aiming to reduce nuisance and protect public health. Concurrently, there was a successful effort to limit the number of mosquitoes expected to overwinter in the upcoming period. Furthermore, supplemental ground-based ultra-low volume (ULV) adulticiding applications - referring to the practice of using chemical or biological agents to kill adult insects - were carried out to enhance the effectiveness of the control measures. The number of carbon dioxide traps (CDC) increased from ten to twelve to improve the monitoring of the adult mosquito population, particularly in light of the rising migratory flows in certain areas. Figure 2 illustrates the spraying carried out in 2017, implementing the mosquito control program, and analyzing each type of spraying in relation to each field. It is clearly shown that the larvicidal interventions covered 37.5% of the annual total interventions anticipated by the tender, while the percentage of residual adulticiding interventions covered 60% of the projected interventions (Figure 2).

**Figure 3:**
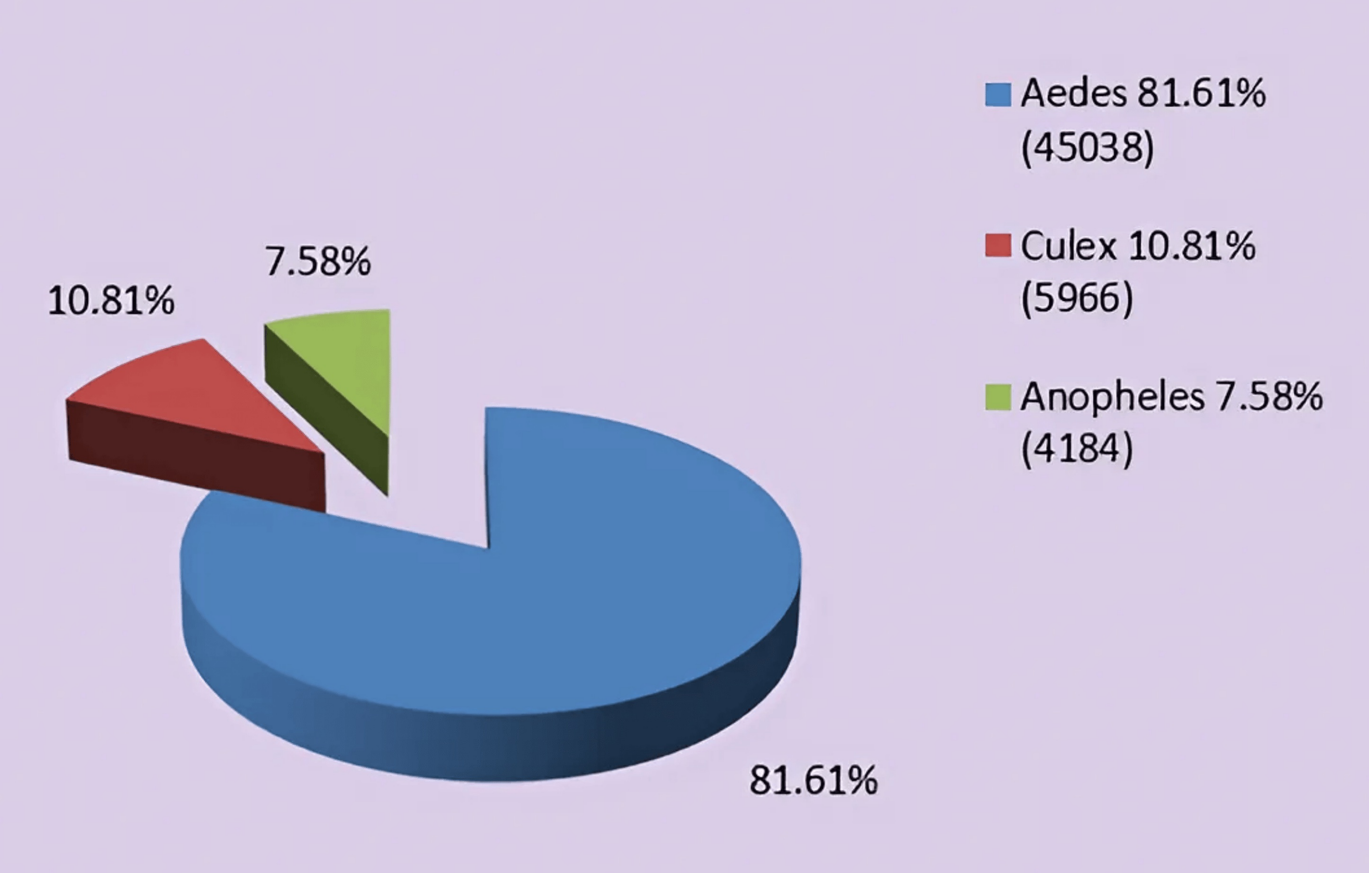
Percentage representation and numbers of the populations of the three dominant genres for the year 2017. Image credits: Nanos CF, Lialiaris TS, authors

**Figure 4:**
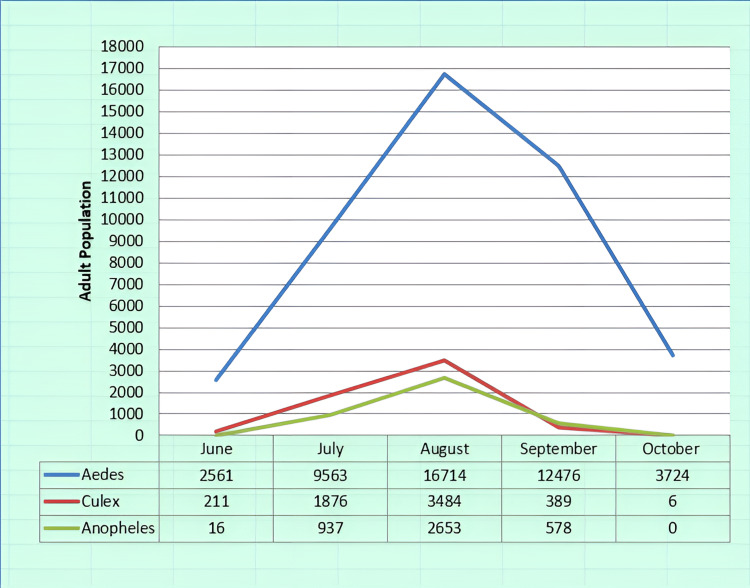
Presence of dominant genre by month for the year 2017. Image credits: Nanos CF, Lialiaris TS, authors

Figure 3 analyzes the three dominant mosquito species in 2017, collected in the active mosquito traps, and their percentages to show their health significance. In the analysis for the time period concerning the first year of the program (27/07/2017 - 20/10/2017), we included data from two samples collected under the previous program to obtain a more accurate and reliable picture of the evolution of population density in the area, as well as the species (Figures 3-4). 

Furthermore, Figure 4 presents in detail the escalation during 2017 of the mosquito control program for the three dominant species to understand how and when efforts to combat them should be intensified.

In 2017, 48 diagnosed cases of the West Nile virus were recorded in Greece, with no cases reported in the Evros region. Conversely, seven cases were reported in neighboring Turkey and one in Bulgaria, while other cases were noted in Italy, Serbia, Romania, Croatia, Israel, France, and Hungary. It is evident that this virus has established itself in Greece, and the circulation and appearance of cases are considered almost certain in both known and new areas, due to the complex epidemiology of the virus, making its circulation unpredictable [10]. 

All three mosquito varieties showed an increasing trend in their occurrence during the first three months of the recording year 2017 (*Aedes*: by 7080 mosquitoes per month; *Culex*: by 1640 mosquitoes per month; *Anopheles*: by 1320 mosquitoes per month). The maximum number of mosquitoes for all three varieties was observed in the month of August (*Aedes*: 16714 mosquitoes; *Culex*: 3484 mosquitoes; *Anopheles*: 2653 mosquitoes). Subsequently, all three mosquito varieties showed a decreasing trend in their occurrence during the following months of the recording period (*Aedes*: by 6500 mosquitoes per month; *Culex*: by 1740 mosquitoes; *Anopheles*: by 1325 mosquitoes per month).

Overview of the situation in 2018

**Figure 5:**
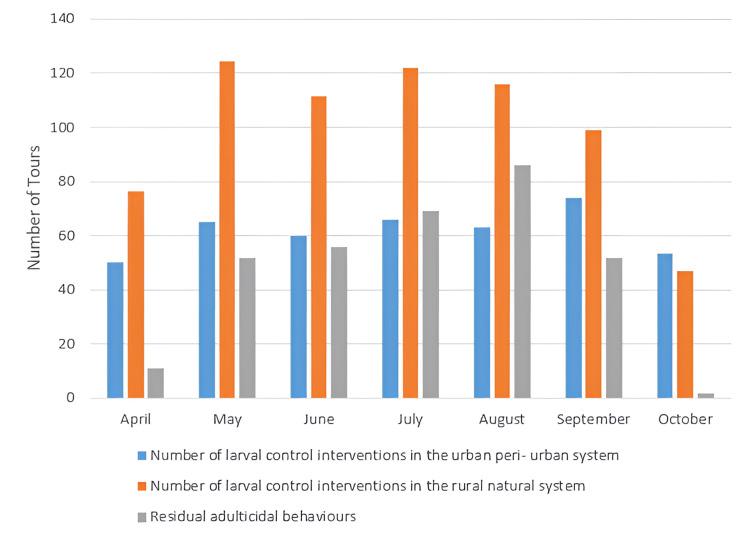
Graphical representation of spraying interventions for 2018. Image credits: Nanos CF, Lialiaris TS, authors

For 2018, a valid start of the control program occurred, which began in April and ended in October. The intensity of applications was proportional to the prevailing conditions in the area, along with field findings collected from the network of traps (12) - CDC, in conjunction with larval sampling and the identification of *West Nile virus *and malaria cases (Figure 5,7). Drones were experimentally used in certain areas within the natural and peri-urban system, where there were significant access difficulties and an increased number of stagnant waters. However, there were several challenges in obtaining the necessary permits for using these modern technical means. 

**Figure 6:**
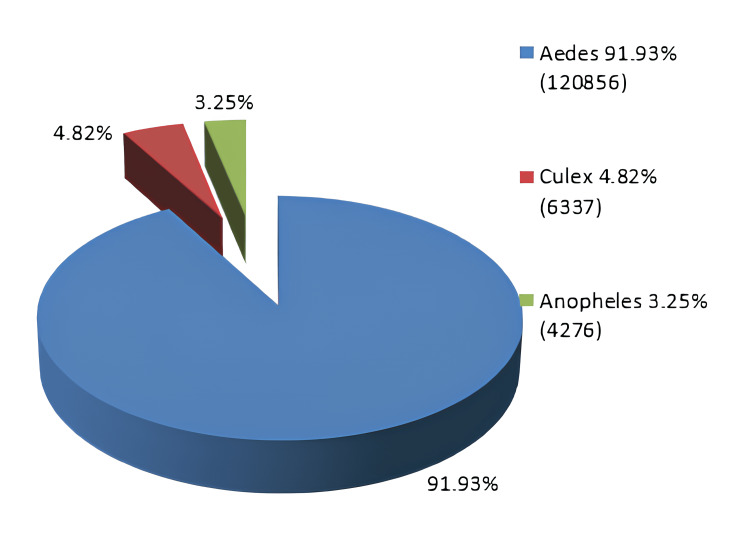
Percentage graphical representation and numbers of the dominant mosquito genres for the year 2018. Image credits: Nanos CF, Lialiaris TS, authors

**Figure 7:**
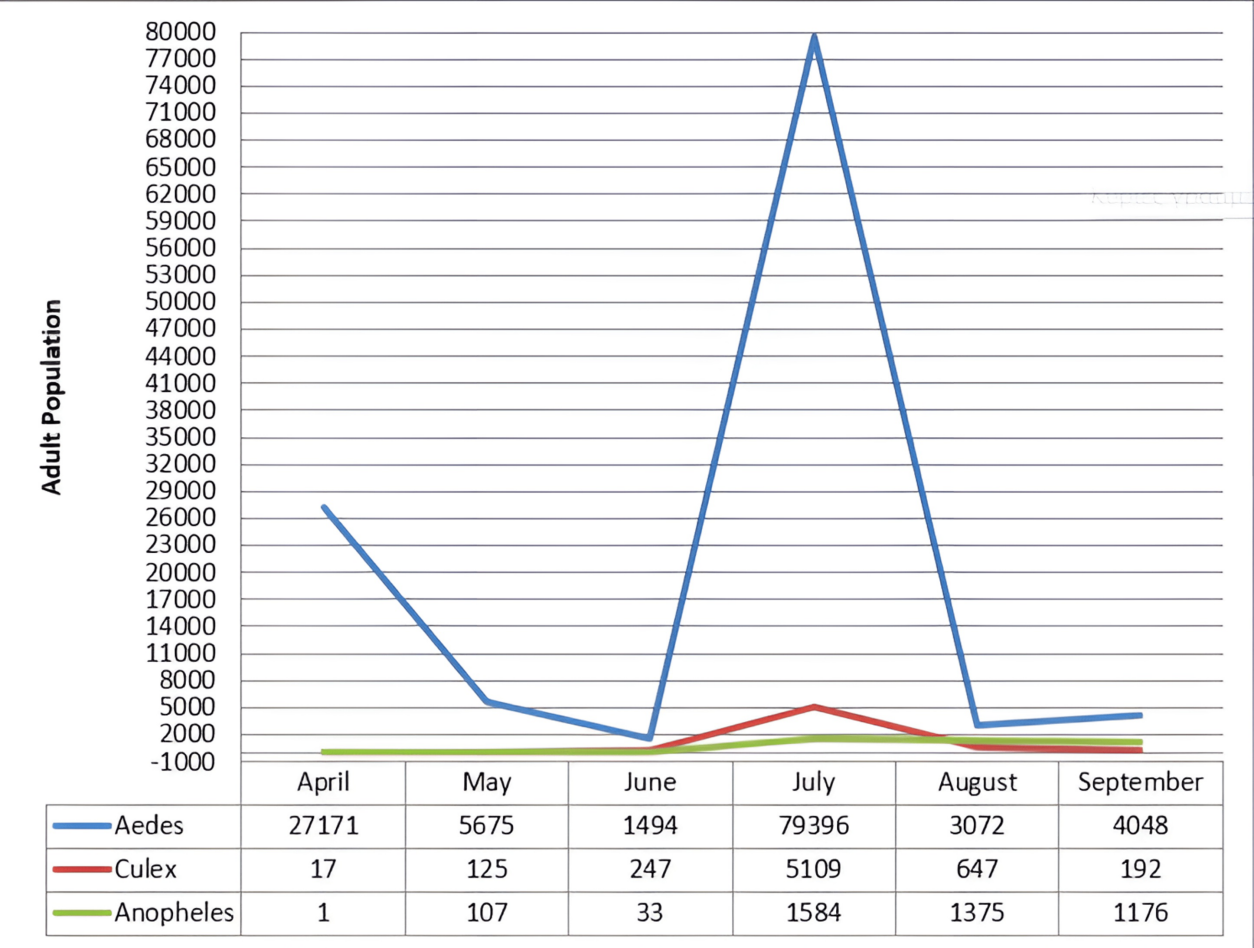
Presence of dominant genres by month for the year 2018. Image credits: Nanos CF, Lialiaris TS, authors

Figure 5 shows the spraying carried out in 2018, implementing the mosquito control program, and analyzing each type of spraying in relation to each field. Figure 6 analyzes the three dominant mosquito species 2018 collected in the active mosquito traps and their percentages to show their health significance. Finally, Figure 7 presents in detail the escalation during 2018 of the mosquito control program for the three dominant species to understand how and when efforts to combat them should be intensified.

For the year 2018, the *West Nile virus* made an early appearance, with an increased number of cases in Greece and across Europe. In our country, 316 domestic cases were recorded, along with one imported case, which had a travel history from another European country. In the Evros region, five cases were recorded. The entire European Union and neighboring countries experienced an increased number of cases compared to previous years. The total number rose to 2.083, surpassing the total number of cases in the past seven years. Neighboring Turkey reported 23 cases, Bulgaria 15, Italy 550, Israel 110, France 24, Cyprus, Croatia, Kosovo, Czech Republic, Romania, and Slovenia [10]. Epidemiological data for malaria in the Evros region showed 2 patients with indications of local transmission. One patient had a probable exposure location in the Tychero municipal unit of Soufli municipality, and the other was in a settlement within the Ferres municipal unit of Alexandroupolis municipality. Of note, in another settlement of the same municipal unit, two local malaria cases were recorded in 2013 [14]. 

During the first three months of the recording year 2018 (April to June), a downward trend was observed in the number of *Aedes* mosquitoes (by 12,840 mosquitoes per month), which showed a significant increase in the month of July. The other two mosquito varieties showed an increasing trend in their occurrence during the first four months of the recording period (*Culex*: by 1,540 mosquitoes per month; *Anopheles*: 470 mosquitoes per month). The maximum number of mosquitoes for all three varieties was observed in the month of July (*Aedes*: 79,396 mosquitoes; *Culex*: 5,109 mosquitoes; *Anopheles*: 1,584 mosquitoes). Subsequently, all three mosquito species showed a downward trend in their occurrence during the following months of the recording period (*Aedes*: by 37,670 mosquitoes per month; *Culex*: by 2,460 mosquitoes; *Anopheles*: by 200 mosquitoes per month).

Furthermore, a statistically significant difference was observed in the distribution (frequency of occurrence) of the three dominant mosquito species in the years 2017 versus 2018 (X^2^ = 4,188.93, df = 2, p < 0.001). More specifically, the *Aedes* variety appeared more frequently in 2018, while the *Culex* and *Anopheles* varieties appeared more frequently in 2017.

## Discussion

One of the main goals of this work is that the results of the study will awaken the medical community so that it can diagnose diseases in a timely manner (e.g., malaria, etc.), which is why this particular journal was chosen. At the same time, people who plan the strategic policy to combat mosquitoes will extract information that there is a large appearance of mosquitoes of the genus *Aedes*, which previously did not exist in such large populations. This particular species of mosquito is responsible for causing significant problems in public health.

Mosquito nuisance levels, particularly in the Evros region, remain high, especially in Northern Evros. In agricultural areas where rice paddies exist and in the Delta of the Evros River, combined with particularly hot and humid summer months (2015, 2016, 2017, and 2018), contribute to the ideal conditions for the emergence and spread of epidemics via mosquitoes (cases of West Nile virus). The implementation of mosquito control programs needs changes to be improved. The lack of cross-border coordination in conjunction with climate change, as well as the lack of tools like a centralized reporting platform for mosquito species and their evolution, causes scientists to worry and refer to a "public health time-bomb," especially in relation to the illegal migration that has emerged in recent years [10,15].

There is an urgent need for quicker modernization and restructuring of the current control program, such as using sentinel bird cages, and collaborations between the three neighboring countries, i.e., Greece, Turkey, and Bulgaria, to establish joint control bodies or information exchange programs [16]. In addition, the incorporation of new control methods utilizing modern tools such as drones is essential [12-13,17]. It has become evident that the effectiveness of larviciding as a control program will be limited when not being accompanied by other methods such as adulticiding [18-19]. The high number of adults captured in traps, thereby validating the success or failure of the control program, confirms this observation [10,20-21] (Figures 4, 7).To evaluate the results, the final life stage of the mosquitoes should be studied, as this is when the adult mosquitoes have developed [22-23]. Therefore, if a reduction in the population of adult mosquitoes is observed after the application of larviciding, it indicates that our method was effective [24]. At the same time, it would be beneficial to implement certain measures to limit the intensity and frequency of control actions, as well as the cost of the overall program. These measures should focus on the careful management of larval breeding sites and adult overwintering habitats [25] (Figures 3, 5). In conjunction with the above, maintenance of the irrigation system, cleaning of reed beds around irrigation channels, and sealing of cesspools and wells should be included. The program will be considered "complete" when it can adapt over time [1,9,26], specifically when it can align with scientific and technological advancements, and primarily when it can synchronize with the peculiarities of situations, such as the biology of mosquitoes and technical constraints for the respective application area [10,27]. The difficulties with larger-scale projects include (a) inaccessible breeding sites; (b) large-scale breeding sites; (c) presence of dense vegetation covering large areas, such as rice fields; (d) occurrence of epidemics in the region transmitted by mosquitoes, such as the West Nile Virus and Malaria; (e) weather conditions; (f) large number of breeding sites, which vary over time; and (g) presence of breeding sites in protected areas (Natura). 

An integrated mosquito control program comprises a series of actions carried out throughout the year. The establishment of a targeted timeline for the implementation of various actions is crucial, as certain interventions must occur at the appropriate time [12,21,25] (Figures 2, 5). Just before the end of the winter season, it is necessary to reassess potential breeding sites and areas where adults may overwinter, as well as update electronic records of these sites as databases. Special attention should be given to the species *Culex*, which is a significant vector of diseases. There is also the possibility of mixed populations with indigenous, non-indigenous, and hybrid mosquitos. Indigenous populations remain active throughout the winter, showing low survival rates due to particular seasonal conditions, while non-indigenous populations enter hibernation. Therefore, interventions for urban control should begin towards the end of winter and the beginning of spring, targeting specific areas to limit their numbers [6,24,27]. 

In the springtime, specifically in April, monitoring of the adult mosquito network begins. According to data collected from our databases, combined with existing literature, it has been found that the activity of *Aedes* species is more pronounced than that of other species. *Culex* and *Anopheles *species appeared to a lesser extent, with their activity peaking during the summer [1,5,9,16]. It was also observed that when an area experiences heavy rainfall for several days and the temperature hovers around 20°C, ideal conditions for *Aedes* egg hatching and the development of larvae into adult mosquitoes are created [1,3,16]. The economic and operational fragmentation of resources and programs based on administrative boundaries inherently reduces the expected effectiveness of projects, as the nature of the problem, from a scientific perspective, depends on different types of boundaries, such as geomorphological and transnational ones. Thus, there is a risk that resources may not be utilized in the most efficient manner, and consequently, this distribution may overlook the characteristics that define the nature of the issues [11,19,28].

A thorough understanding of the ecology of mosquito species endemic to an area is quite important for designing effective strategies for action and population control [24,26]. Breeding sites found in large and open water areas, often caused by floods, differ in management from those in urban and peri-urban environments. They may pose significant public health problems, with the *Aedes *and *Culex* *pipiens* species being the most important in this category [28-29]. Urgent adulticide treatment is recommended if increased nuisance levels are detected compared to previous data. The larvicidal formulations used do not cause toxicity to other beneficial insects, including bees. They are applied only to water collections and if there are larvae in them. During this process, especially bees, do not remain in the spraying area. At the same time, the approved mosquito control preparations are basically photodegradable. Therefore, during the day and after they have achieved their goal, the elimination of mosquitoes, they cease to exist.

The first appearance of *Aedes *albopictus in Greece was recorded in Rhodes in 2003. Since 2015, it has also been observed in the Evros region, causing small outbreaks to this day. This situation is quite concerning due to the transmission of infectious diseases by this particular species [1,3,9,16-17,30]. Therefore, it has become necessary to deploy traps, which are set monthly for a duration of seven days. 

## Conclusions

Mosquito control programs, organized by the state or local authorities, are inherently flexible and evolve over time as the problem they address changes. It is essential that these programs keep up with advancements in science, technology, and new knowledge regarding the specific conditions of each area. Unfortunately, different factors can create challenges in the implementation of these programs, affecting their efficiency. Within the framework of collaborations and a future national plan for the prevention of mosquito-borne diseases, it is vital to emphasize additional cross-border and well-structured plans. Mosquitoes do not recognize borders or ownership titles and, unfortunately, intrude, creating significant problems for public health.

Some proposals for cross-border coordination in mosquito control could include (a) the development of a joint strategic plan, where every country affected by the mosquito problem should collaborate in developing a unified strategic plan; (b) joint monitoring and data collection, where cooperation in monitoring mosquito populations and related diseases (e.g., West Nile virus, Malaria) is crucial, and shared databases could enable continuous recording and analysis of mosquito levels and geographical distribution; (c) joint research initiatives, where countries can collaborate on research focusing on the development of innovative methods for mosquito control; (d) coordinated information and education programs, where countries could develop joint educational programs and awareness campaigns that include basic practices such as the removal of standing water from urban areas; (e) exchange of expertise and best practices, where the experience and expertise of diffeent countries can be exchanged and utilized to improve the implementation of control strategies; (f) intergovernmental agreements and funding, where the development of agreements between governments and the international community for funding joint mosquito control efforts; (g) infrastructure development projects, where collaboration on upgrading infrastructure, such as constructing drainage systems or improving sanitation, can reduce mosquito populations, particularly in areas frequently affected by floods; and finally (h) coordinated action in border areas, where coordination should be intensified in areas near borders, as mosquitoes do not recognize geographical boundaries.

As a final conclusion, we can highlight that all measures taken need to consider always the environmental impact, safeguarding the environment, the general biodiversity, and human health.

## References

[1] Badieritakis Ε, Papachristos D, Latinopoulos D (2018). Aedes albopictus (Skuse, 1895) (Diptera: Culicidae) in Greece: 13 years of living with the Asian tiger mosquito. Parasitol Res.

[2] Samanidou A, Harbach RE (2001). Keys to the adult female mosquitoes (Culicidae) of Greece. Eur Mosq Bull.

[3] Samanidou-Voyadjoglou A, Patsoula E, Spanakos G, Vakalis NC (2005). Confirmation of Aedes albopictus (Skuse) (Diptera: Culicidae) in Greece. Eur Mosq Bull.

[4] Tsiodras S, Pervanidou D, Papadopoulou E (2016). Imported Chikungunya fever case in Greece in June 2014 and public health response. Pathog Glob Health.

[5] Marka A, Diamantidis A, Papa A (2013). West Nile virus state of the art report of MALWEST Project. Int J Environ Res Public Health.

[6] ECDC: VBORNET. . https://www.eea.europa.eu/data-and-maps/data/external/network-of-medical-entomologists-and (2025). ECDC: VBORNET vector distribution maps. https://www.eea.europa.eu/data-and-maps/data/external#c0=10&c7=all&b_start=0.

[7] ECDCa: (2012 (2013). ECDC: Joint WHO-ECDC mission related to local malaria transmission in Greece, 2012. https://www.ecdc.europa.eu/en/publications-data/joint-who-ecdc-mission-related-local-malaria-transmission-greece-2012.

[8] N.P.H.O./E.O.D.Y.: (2023 (2023). N.P.H.O.: annual epidemiological reports-malaria in Greece. https://eody.gov.gr/wp-content/uploads/2024/06/malaria_annual_report_2023_eng.pdf.

[9] Giatropoulos A, Michaelakis A, Koliopoulos G, Pontikakos C (2012). Records of Aedes albopictus and Aedes cretinus (Diptera: Culicidae) in Greece from 2009 to 2011. Hell Plant Protection J.

[10] N.P.H.O./E.O.D.Y.: (2010-19 (2019). N.P.H.O.: (2010-19). West Nile virus. General Information. https://eody.gov.gr/disease/ios-toy-dytikoy-neiloy/?utm_medium=email&utm_source=transaction.

[11] Patsoula E, Vakali A, Balatsos G (2016). West Nile Virus Circulation in Mosquitoes in Greece (2010-2013). Biomed Res Int.

[12] ECDC ECDC (2012). ECDC: Technical report. Guidelines for the surveillance of invasive mosquitoes in Europe. Stockholm. https://www.ecdc.europa.eu/sites/default/files/media/en/publications/Publications/TER-Mosquito-surveillance-guidelines.pdf.

[13] Semenza JC, Zeller H (2014). Integrated surveillance for prevention and control of emerging vector-borne diseases in Europe. Euro Surveill.

[14] N.P.H.O./E.O.D.Y.: (2018 N.P.H.O.: (2018) Annual Epidemiological Surveillance Report. Malaria in Greece. https://eody.gov.gr/wp-content/uploads/2019/04/Annual_Malaria_report_EN_2018_final.pdf.

[15] Gratz NG (2004). Critical review of the vector status of Aedes albopictus. Med Vet Entomol.

[16] Wheeler AS, Petrie WD, Malone D, Allen F (2009). Introduction, control, and spread of Aedes albopictus on Grand Cayman Island, 1997-2001. J Am Mosq Control Assoc.

[17] Bloukou DE, Andreadis SS, Balatsos G (2019). Monitoring invasive insect species in the region of Kavala (Greek). 18th Panhellenic Entomological Congress, Komotini , Greece, October 15-18.

[18] Schreiber ET, Morris CD (1995). Evaluation of public information packets for mosquito source reduction in two Florida cities. J Am Mosq Control Assoc.

[19] Healy K, Hamilton G, Crepeau T, Healy S, Unlu I, Farajollahi A, Fonseca DM (2014). Integrating the public in mosquito management: active education by community peers can lead to significant reduction in peridomestic container mosquito habitats. PLoS One.

[20] Betzios B (1989). Arthropods of sanitary importance. Morphology, biology, ecology, health importance, control (Greek). Morphology, Biology, Ecology, Health Importance, Control. Athens.

[21] Patsoula E, Beleri S, Vakali A (2017). Records of Aedes albopictus (Skuse, 1894) (Diptera; Culicidae) and Culex tritaeniorhynchus (Diptera; Culicidae) Expansion in Areas in Mainland Greece and Islands. Vector Borne Zoonotic Dis.

[22] Heintze C, Velasco Garrido M, Kroeger A (2007). What do community-based dengue control programmes achieve? A systematic review of published evaluations. Trans R Soc Trop Med Hyg.

[23] Walter Reed Biosystematics Unit.: (2016 (2016). Walter Reed Biosystematics Unit.: Smithsonian Institute. Maryland: silver spring. http:// https://wrbu.si.edu/vectorspecies/keys 2016.

[24] Darsie RF Jr, Samanidou-Voyadjoglou A (1997). Keys for the identification of the mosquitoes of Greece. J Am Mosq Control Assoc.

[25] Becker N, Petric D, Zgomba M, Boase C, Madon M, Dahl C, Kaiser A (2010). Mosquitoes and their control 2nd ed.

[26] Samanidou A, Harbach R (2003). Culex tritaeniorhynchus Giles, a newly discovered potential vector of arboviruses in Greece. Eur Mosq Bull.

[27] Nanos C (2017). Mosquito control programs in the Municipality of Thermaikos from 2003 to 2015 and their contribution to public health master thesis. https://repo.lib.duth.gr/jspui/bitstream/123456789/14280/1/NanosC_2017.pdf.

[28] Petric D, Bellini R, Michaelakis A, Zgomba M (2019). Quality control as a component of mosquito management - do we need it? (Greek). 18th Panhellenic Entomological Congress, Komotini, October 15-18, Greece.

[29] Balatsos G, Karras V, Mastronikolos G (2019). Sterile Insect Technique (SIT) against Aedes albopictus (Diptera: Culicidae). (Greek). 18th Panhellenic Entomological Congress, Komotini, October 15-18, Greece.

[30] Enserink M (2010). Science and society. GM mosquito trial alarms opponents, strains ties in Gates-funded project. Science.

